# Supplementary material for: Orally available designed miniproteins inhibit enterotoxigenic Bacteroides fragilis pathology by blocking toxin receptor binding
Source: bioRxiv. 2026 Jun 23:2026.06.22.733822. Preprint. [Version 1] doi: 10.64898/2026.06.22.733822 (PMC13320741; doi:10.64898/2026.06.22.733822)
Supplement: Supplement 5 [file NIHPP2026.06.22.733822v1-supplement-5.pdf]

bioRxiv preprint doi: https://doi.org/10.64898/2026.06.22.733822; this version posted June 23, 2026. The copyright holder for this preprint (which was not certified by peer review) is the author/funder, who has granted bioRxiv a license to display the preprint in perpetuity. It is made available under a CC-BY 4.0 International license.

Supplemental Figure Legends

Supplemental Figure 1. Yeast surface display-based screening of minibinders designed against the BFT-1 active site.

A-C) Pseudocolor density plots of FITC and PE fluorescence from round one (A) and round two (B) yeast display minibinder library enrichment with exposure to 1 mM BFT-1 (right) or the control treatment, and round three of enrichment (C) which employed a titration of BFT-1 from 1 nM to 1 mM. D) Relative enrichment of yeast clones encoding the indicated minibinders from the third FACS-based enrichment for BFT-1 binders (1 nM BFT-1 sample), as determined by library sequencing read counts.

Supplemental Figure 2. First generation BFT-1 minibinders enriched from yeast surface display are predicted to interact with two surfaces of BFT-1.

A-E) AF3 models for complexes formed by BFT-1 and the indicated minibinders, colored by predicted local distance difference test (pLDDT) values. Corresponding predicted aligned error plots shown underneath each model.

Supplemental Figure 3. Viability of HT-29 cells is unaffected by ETBF spent culture supernatant, and the BFT inhibitory activity of PIBs is specific.

A) Viability of HT-29 cells (as assessed by MTT assays) exposed SCS derived from the indicated strains, or media alone B,C) E-cadherin detected in the cell supernatant from HT-29 cells exposed to minibinders alone, ETBF SCS, or both (B), or ETBF and increasing concentrations of negative control (NC) minibinder designed against a different target (C). Data represent means and standard errors. Asterisks in B indicate minibinder treatments significantly

bioRxiv preprint doi: https://doi.org/10.64898/2026.06.22.733822; this version posted June 23, 2026. The copyright holder for this preprint (which was not certified by peer review) is the author/funder, who has granted bioRxiv a license to display the preprint in perpetuity. It is made available under a CC-BY 4.0 International license.

different from ETBF SCS treatment alone (1-way ANOVA with Dunnett's multiple comparisons test, *p<0.01, **p<0.001, ***<0.0001).

Supplemental Figure 4. Yeast surface display-based screening of minibinders designed against the prodomain interface of BFT-1.

A-F) Pseudocolor density plots of FITC and PE fluorescence from <66 residue design library (A-C) and the 66-88 residue design library (D-F) after one (A,D) or two (B,E) rounds of exposure to 1 mM BFT-1 (right) or the control treatment (left) or third enrichment employing a titration of BFT concentrations (C,F). G) Relative enrichment of yeast clones encoding the indicated minibinders from the third FACS-based enrichment for BFT-1 binders (100 nM BFT-1 sample), as determined by library sequencing read counts.

Supplemental Figure 5. Second generation minibinders are predicted to interact with the prodomain of BFT-1

A-E) AF3 models for complexes formed by BFT-1 and the indicated minibinders, colored by predicted local distance difference test (pLDDT) values. Corresponding predicted aligned error plots shown underneath each model.

Supplemental Figure 6. Second generation BFT inhibitors perform comparably to PIB MB03

A-C) AF3 models of the BFT-1 (white, surface display) interaction with second generation minibinders that passed initial screening criteria but performed poorly in one or more experimental assay. D, E) SPR analysis of the binding affinity of the indicated minibinders for

bioRxiv preprint doi: https://doi.org/10.64898/2026.06.22.733822; this version posted June 23, 2026. The copyright holder for this preprint (which was not certified by peer review) is the author/funder, who has granted bioRxiv a license to display the preprint in perpetuity. It is made available under a CC-BY 4.0 International license.

immobilized BFT-1. K_D values indicate binding affinity calculated from this analysis. F,G) E-cadherin detected in the cell supernatant from HT-29 cells exposed to the indicated purified minibinders alone (F), with ETBF SCS (G) or the indicated controls. H) HT-29 viability (MTT) assays of cells exposed to the indicated purified minibinders. I) Counts of separated, Giemsa-stained HT-29 cells exposed to the indicated minibinders and ETBF SCS or a vehicle control.

Supplemental Figure 7. PIBs prevent BFT-1-mediated murine colonoid damage.

A, B) Murine colonoid integrity following treatment with purified BFT-1 (100 nM) with the indicated minibinders (10 mM) (A), or the indicated minibinders alone (B).

Supplemental Figure 8. Binding assays support predicted MB03-BFT-1 interaction residues, and PIBS interfere with the BFT-claudin-4 interaction.

A) Biolayer interferometry analysis of the binding affinity of BFT-1 for immobilized MB03 variants containing the indicated substitutions. K_D values indicate binding affinity calculated from this analysis. Colored traces indicate measured data; black curves indicate corresponding fitted data. B) AF3 model of BFT-1 (surface) to claudin-4 (ribbon, grey), with and without the indicated minibinder. Steric clashes are defined using the MolProbity criterion (interatomic distance is less than the sum of their van der Waals radii minus 0.4 Å) and are shown in red. C) Predicted aligned error plot of BFT-claudin-4 interaction model. D) Sequence alignment of the BFT-1 and BFT-2 catalytic domains. Prodomain interface (purple) and non-conserved (red) residues indicated. E) Western blot analysis of proteins extracted from WT or claudin-4 KO HT-29 cells alone (control) or incubated with BFT-2–H_6 (5 nM) and the indicated PIB (500 nM).

bioRxiv preprint doi: https://doi.org/10.64898/2026.06.22.733822; this version posted June 23, 2026. The copyright holder for this preprint (which was not certified by peer review) is the author/funder, who has granted bioRxiv a license to display the preprint in perpetuity. It is made available under a CC-BY 4.0 International license.

Supplemental Figure 9. Optimization of assays conditions to detect catalytic activity of BFT-1.

A-C) Coomassie-stained SDS-PAGE analysis of cleavage products generated from actin (2 mM, A, C) or E-cadherin (2 mM, B) incubated with purified BFT-1–H_8 (20 nM), no enzyme (–, negative control), trypsin (20 nM, positive control), or the indicated purified minibinders alone (C) under the noted conditions ($CaCl_2$ supplied at 1 mM where indicated). Minibinders, when added, were supplied at 1 μM.

Supplemental Figure 10. **PIB-secreting strains of *B. ovatus* do not affect ETBF or HT-29 viability in co-culture assays**.

A,B) Released E-cadherin detected in the cell supernatant from HT-29 cells exposed to the indicated live bacteria for five hours (A) or bacterial co-cultures added at a 1:1 ratio (B). MB03 (1 μM) treatment was included for comparison. C) C.f.u counts of bacteria from one of the replicate samples of the experiment shown in (B). Dark grey, ETBF; light grey *B. ovatus*–empty or *B. fragilis*–empty; orange, MB03-secreting strains. Adjacent bars indicate populations measured in the same mouse. ETBF populations could not be assessed in the *B. fragilis*-empty colonized mouse due to a technical error. D) Viability (MTT) assays of HT-29 cells treated with the indicated live bacteria. E, F) C.f.u. counts of the bacterial inoculum (E) or post incubation with HT-29 (F). Bacteria were administered at the indicated initial ratios (ETBF:*B. ovatus*). Counts correspond to cultures assayed for E-cadherin release in Fig. 4D.

Supplemental Figure 11. PIB-secreting *B. ovatus* strains prevent ETBF-mediated TER reductions when applied to confluent cultured epithelial cells.

bioRxiv preprint doi: https://doi.org/10.64898/2026.06.22.733822; this version posted June 23, 2026. The copyright holder for this preprint (which was not certified by peer review) is the author/funder, who has granted bioRxiv a license to display the preprint in perpetuity. It is made available under a CC-BY 4.0 International license.

A) C.f.u counts of bacteria applied to the apical surface of HT-29-MTX-E12 cells grown in transwells for conducting TEER assays. B,C) TEER readings of HT-29-MTX-E12 cells treated with either bacterial monocultures (B) or co-culture (C). Co-cultures were added at a 1:1 ratio and incubated for five hours between measurements.

Supplemental Figure 12. Introduction of a stabilizing disulfide bond does not substantially reduce PIB binding affinity or inhibition of BFT-1.

A) AF3 models of the indicated minibinders. B) SPR analysis of the binding affinity of the indicated minibinders for immobilized BFT-1–H_8. K_D values indicate binding affinity calculated from this analysis. C) Released E-cadherin detected in the cell supernatant from HT-29 cells exposed to the indicated purified minibinders. D) SDS-PAGE analysis of the indicated PIBs incubated for the indicated intervals in synthetic gastric or intestinal fluid (SGF, SIF). Asterisks in C indicate minibinder treatments significantly different from ETBF SCS treatment alone (1-way ANOVA with Dunnett's multiple comparisons test, *p<0.0001). Data represent means and standard errors. Asterisks in C indicate minibinder treatments significantly different from ETBF SCS treatment along (1-way ANOVA with Dunnett's multiple comparisons test, * *<0.0001).

Supplemental Figure 13. Mouse cecal injection data

A) Full hematoxylin and eosin (H&E)-stained sections of mouse ceca injected with either PBS (vehicle), MB03-ds, MB15-ds, BFT-2, BFT-2 and MB03-ds, or BFT-2 and MB15-ds. Quantification of cecum histopathology of edema score (B), shedding score (C), and overall score (D), from ceca in (A). (1-way ANOVA with Dunnett's multiple comparisons test, *p<0.05, ***<0.001).

bioRxiv preprint doi: https://doi.org/10.64898/2026.06.22.733822; this version posted June 23, 2026. The copyright holder for this preprint (which was not certified by peer review) is the author/funder, who has granted bioRxiv a license to display the preprint in perpetuity. It is made available under a CC-BY 4.0 International license.

Supplemental Figure 14. Mice are successfully co-colonized with ETBF and *B. ovatus.*

A) C.f.u. counts of bacterial inocula administered to antibiotic pre-treated mice by oral gavage during colonization experiments shown in B. B) Fecal abundance of the indicated strains from mice colonized with ETBF monoculture and *B. ovatus* administered at the indicated ratio of *B. ovatus* to ETBF (0.1-100X). Strains contained antibiotic resistance cassettes (erm and tet, respectively) for quantification by plating on selective media. Graphs show the mean and standard deviation of three to six mice per group.

Supplemental Figure 15. AOM/DSS treated mice with LBPs or PIBs in drinking water have reduced tumor burden.

A) C.f.u. counts of bacterial inoculum administered to mice by oral gavage. B) Mean body weights of mice exposed to the indicated treatments. C) Water consumption of the minibinder-treated mice throughout the experiment. Plots in B and C indicate means and standard errors derived from three to ten mice per group. D) Western blot analysis of VSV-G–MB03 in fecal pellets collected from representative mice the indicated number of days post introduction of *B. ovatus*–MB03 via oral gavage. E) Spectral counts and unique peptides detected by mass spectrometry analysis of proteins associated with Ni-NTA beads incubated with BFT–H_8 (or beads only control, middle) and clarified extracts of pooled fecal pellets collected from mice colonized with ETBF (first column) or colonized with ETBF and supplied with MB03-ds in drinking water (last column). F) Representative hematoxylin and eosin (H&E)-stained colons from the indicated treatment groups. Insets show intact Swiss rolled colons, with the enlarged

bioRxiv preprint doi: https://doi.org/10.64898/2026.06.22.733822; this version posted June 23, 2026. The copyright holder for this preprint (which was not certified by peer review) is the author/funder, who has granted bioRxiv a license to display the preprint in perpetuity. It is made available under a CC-BY 4.0 International license.

regions, representing distal colons, boxed. Black arrows indicate tumors. Inset scale bar = 1 mm; enlargement scale bar = 200 μm.

bioRxiv preprint doi: https://doi.org/10.64898/2026.06.22.733822; this version posted June 23, 2026. The copyright holder for this preprint (which was not certified by peer review) is the author/funder, who has granted bioRxiv a license to display the preprint in perpetuity. It is made available under a CC-BY 4.0 International license.

Supplemental Figure 1

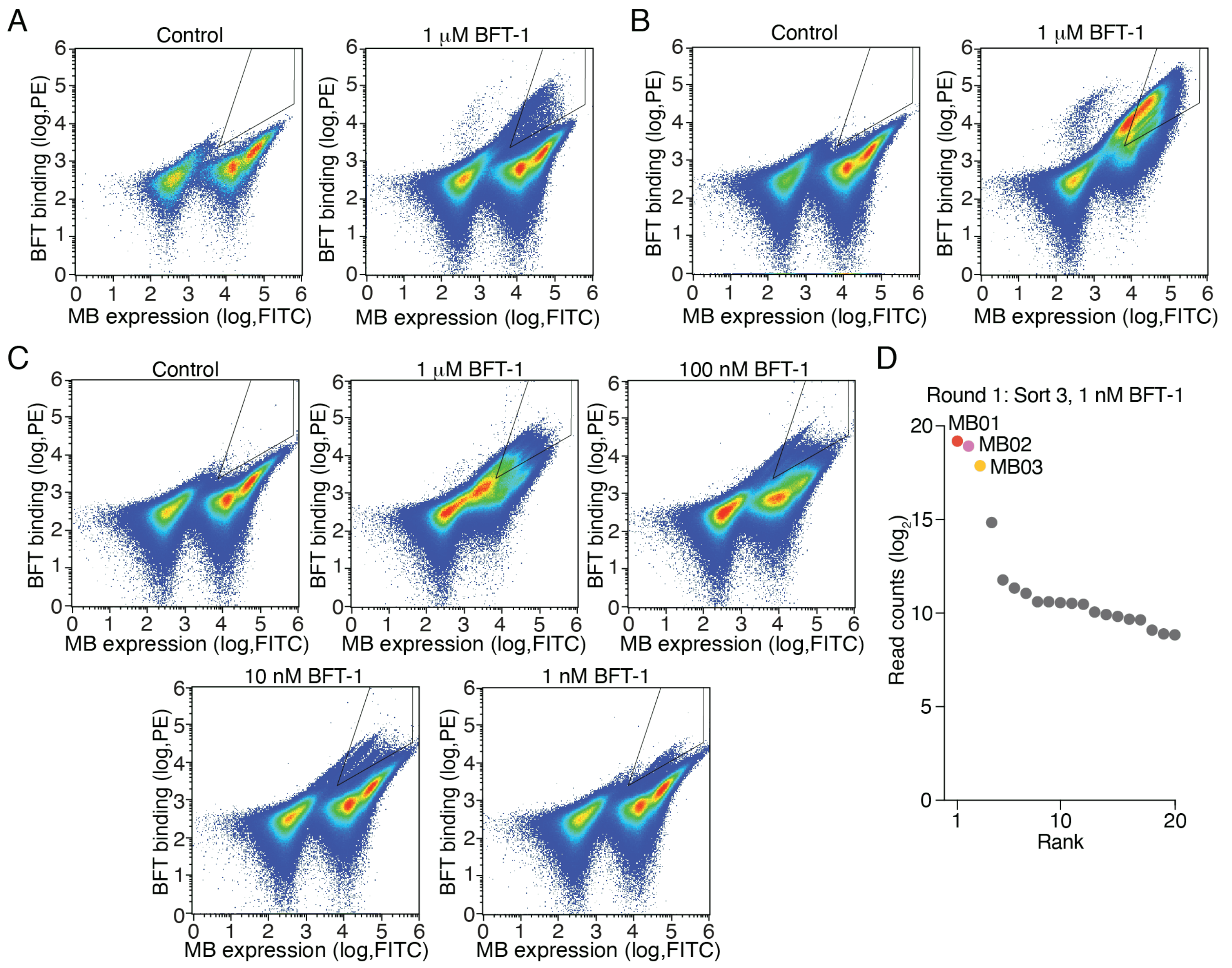

bioRxiv preprint doi: https://doi.org/10.64898/2026.06.22.733822; this version posted June 23, 2026. The copyright holder for this preprint (which was not certified by peer review) is the author/funder, who has granted bioRxiv a license to display the preprint in perpetuity. It is made available under a CC-BY 4.0 International license.

Supplemental Figure 2

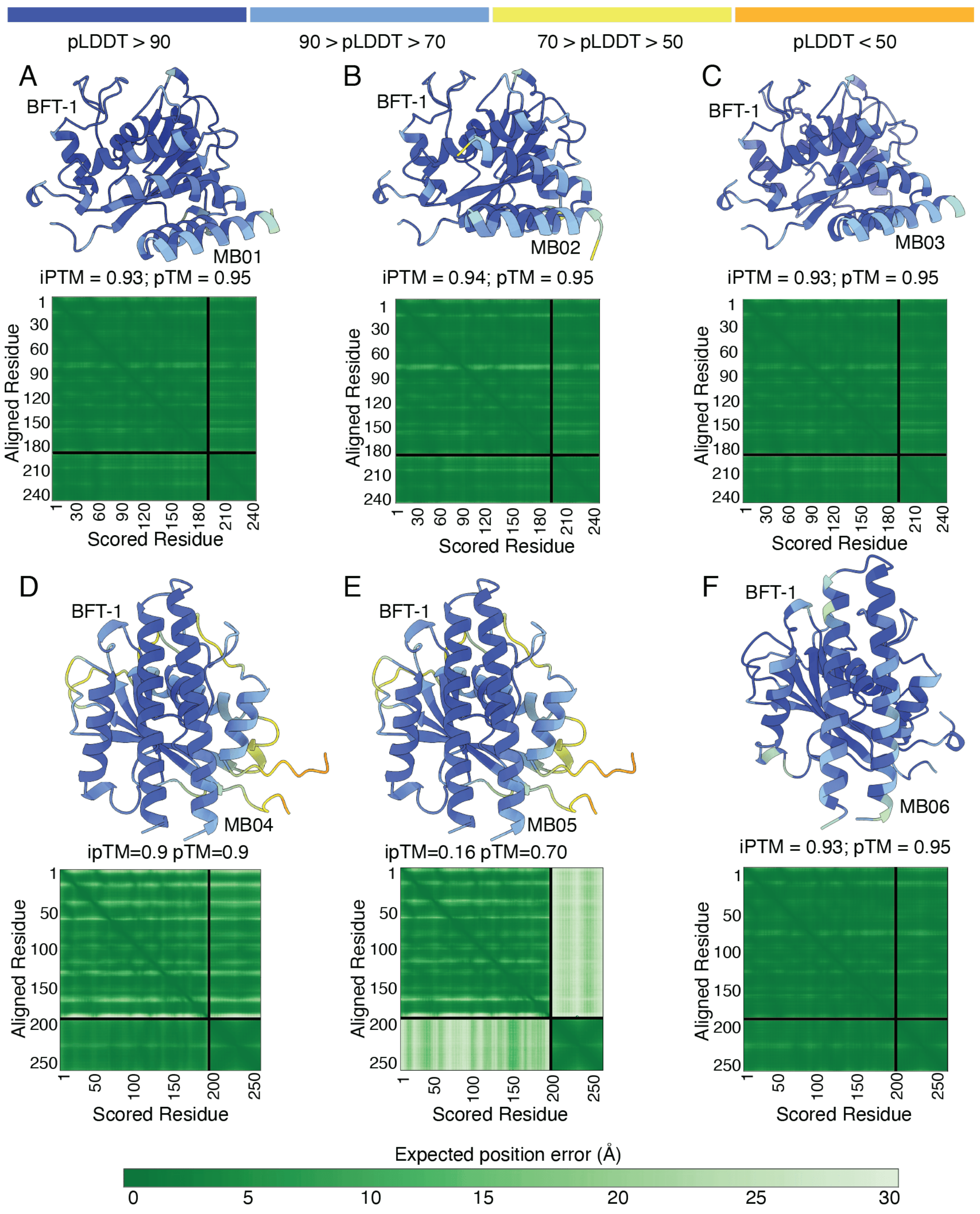

bioRxiv preprint doi: https://doi.org/10.64898/2026.06.22.733822; this version posted June 23, 2026. The copyright holder for this preprint (which was not certified by peer review) is the author/funder, who has granted bioRxiv a license to display the preprint in perpetuity. It is made available under a CC-BY 4.0 International license.

Supplemental Figure 3

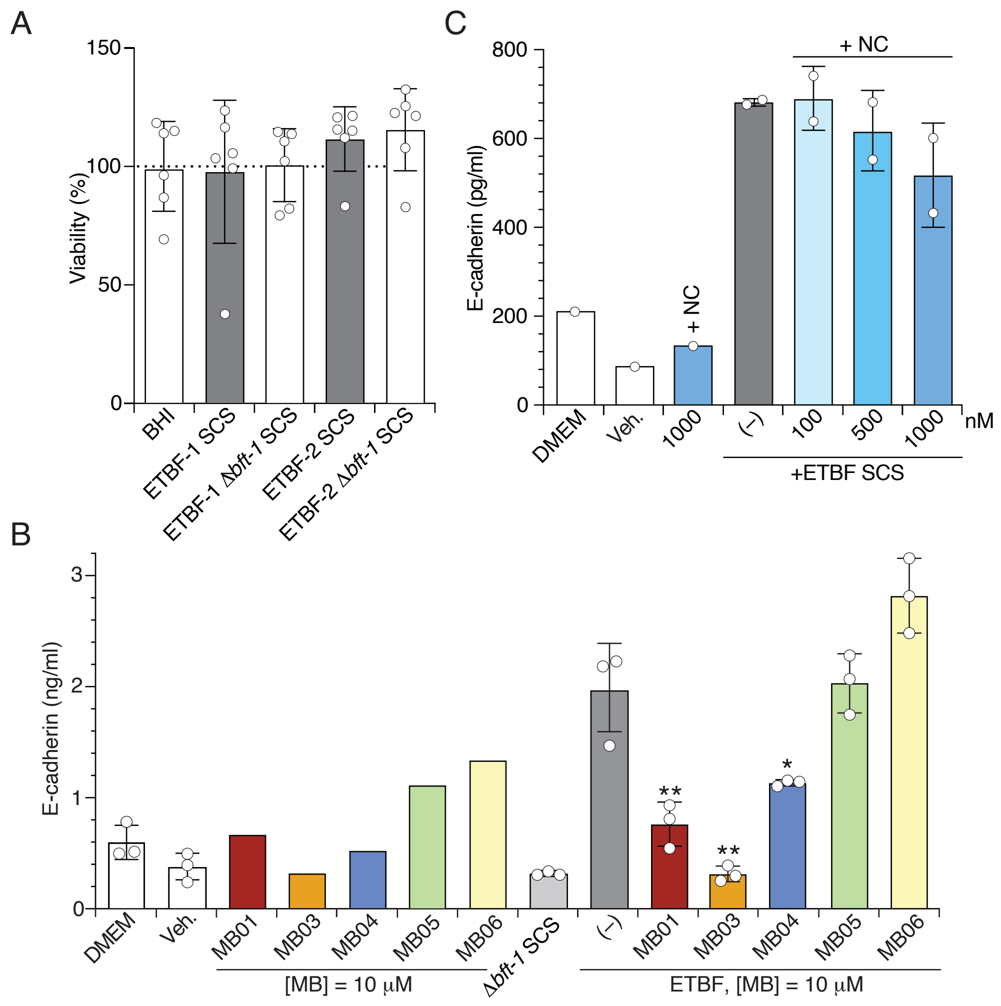

bioRxiv preprint doi: https://doi.org/10.64898/2026.06.22.733822; this version posted June 23, 2026. The copyright holder for this preprint (which was not certified by peer review) is the author/funder, who has granted bioRxiv a license to display the preprint in perpetuity. It is made available under a CC-BY 4.0 International license.

Supplemental Figure 4

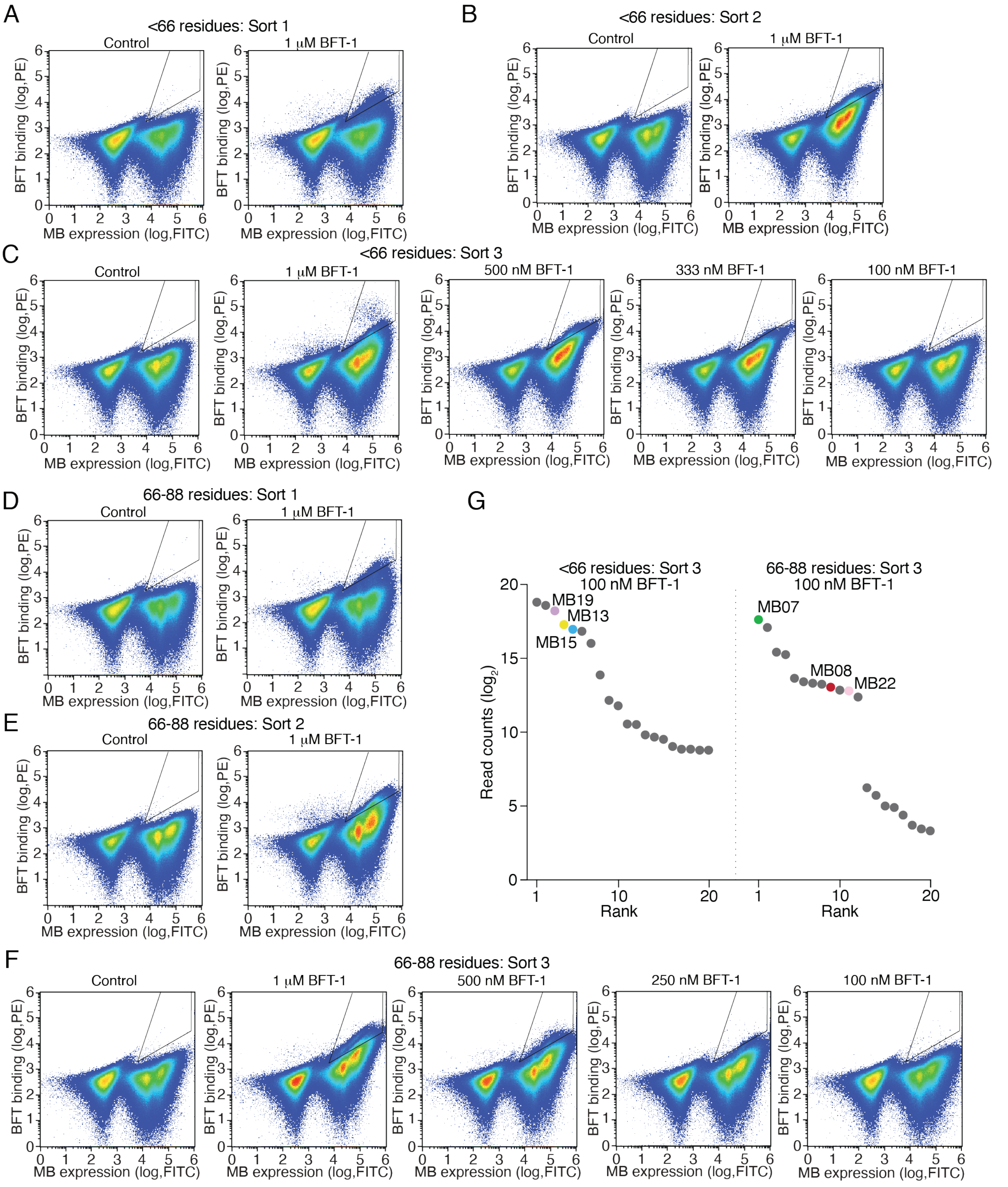

bioRxiv preprint doi: https://doi.org/10.64898/2026.06.22.733822; this version posted June 23, 2026. The copyright holder for this preprint (which was not certified by peer review) is the author/funder, who has granted bioRxiv a license to display the preprint in perpetuity. It is made available under a CC-BY 4.0 International license.

Supplemental Figure 5

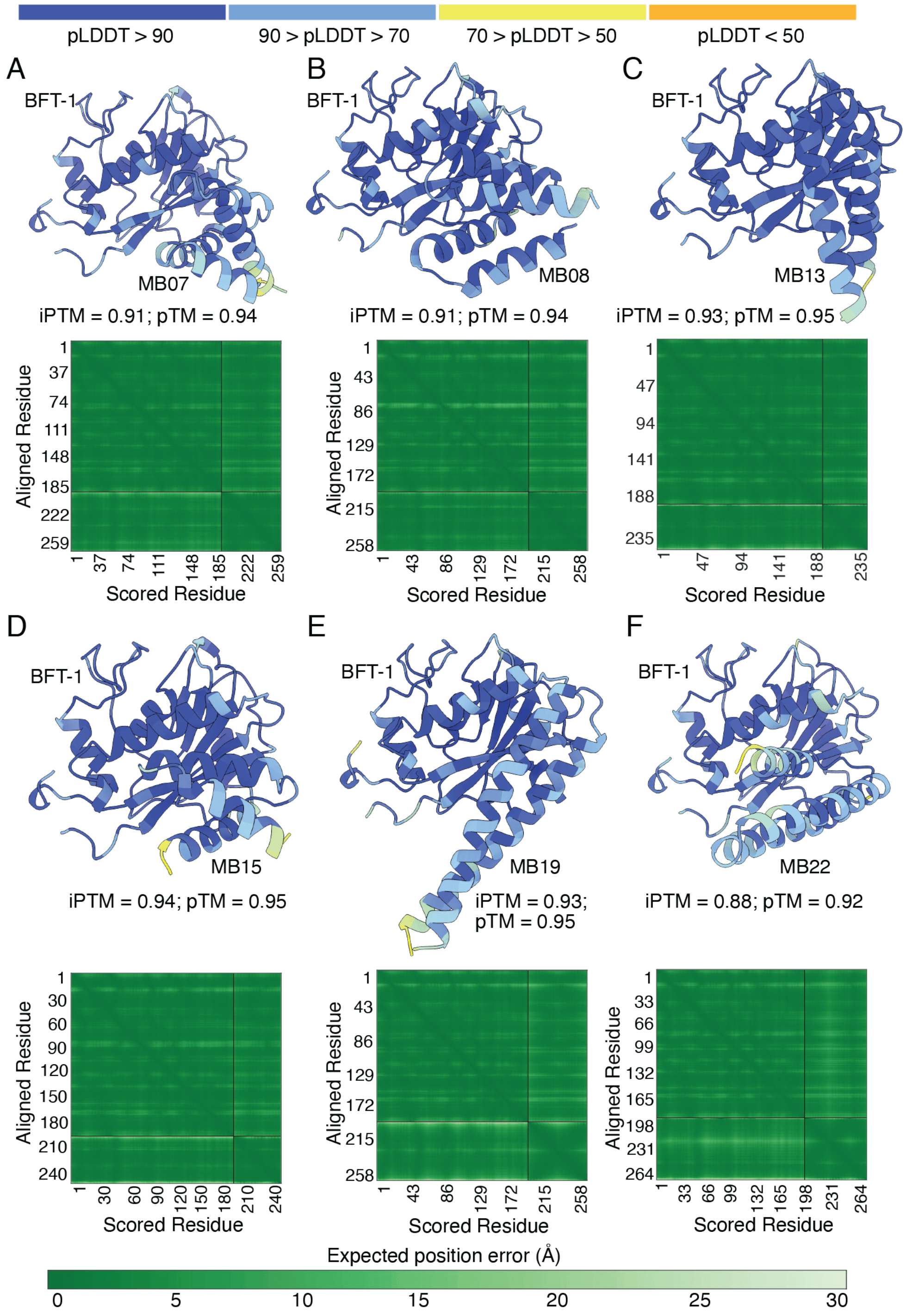

bioRxiv preprint doi: https://doi.org/10.64898/2026.06.22.733822; this version posted June 23, 2026. The copyright holder for this preprint (which was not certified by peer review) is the author/funder, who has granted bioRxiv a license to display the preprint in perpetuity. It is made available under a CC-BY 4.0 International license.

Supplemental Figure 6

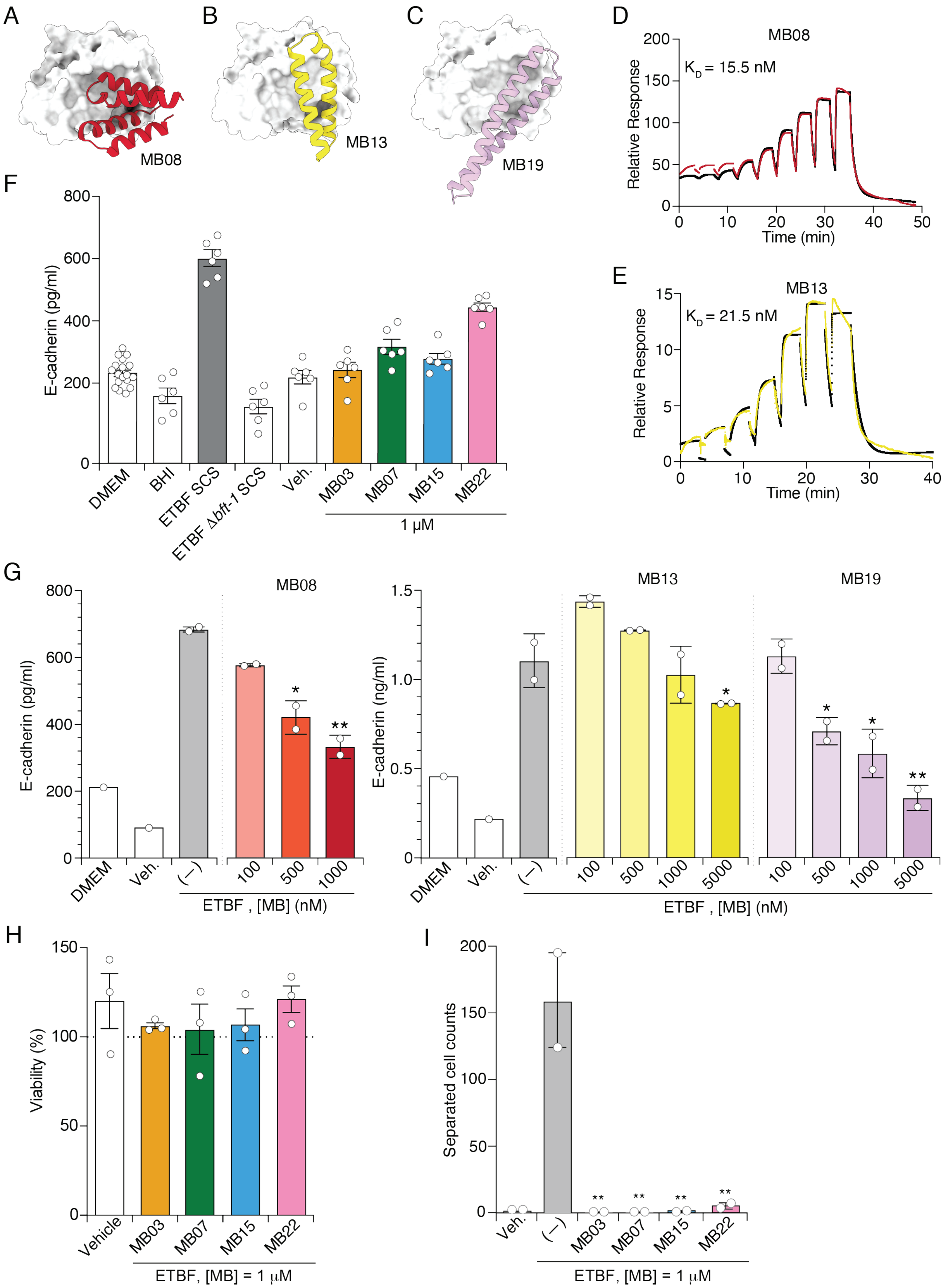

bioRxiv preprint doi: https://doi.org/10.64898/2026.06.22.733822; this version posted June 23, 2026. The copyright holder for this preprint (which was not certified by peer review) is the author/funder, who has granted bioRxiv a license to display the preprint in perpetuity. It is made available under a CC-BY 4.0 International license.

Supplemental Figure 7

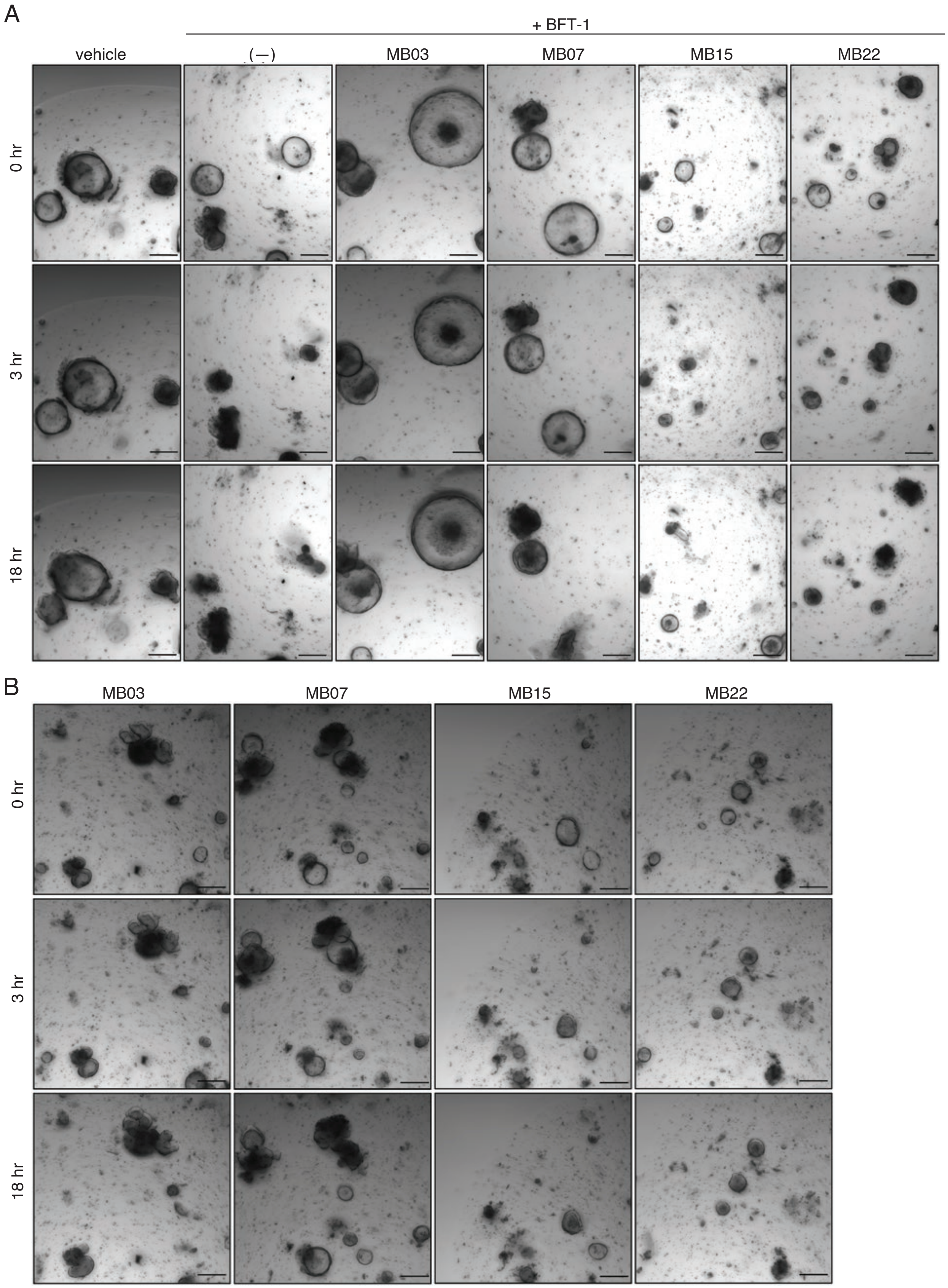

bioRxiv preprint doi: https://doi.org/10.64898/2026.06.22.733822; this version posted June 23, 2026. The copyright holder for this preprint (which was not certified by peer review) is the author/funder, who has granted bioRxiv a license to display the preprint in perpetuity. It is made available under a CC-BY 4.0 International license.

Supplemental Figure 8

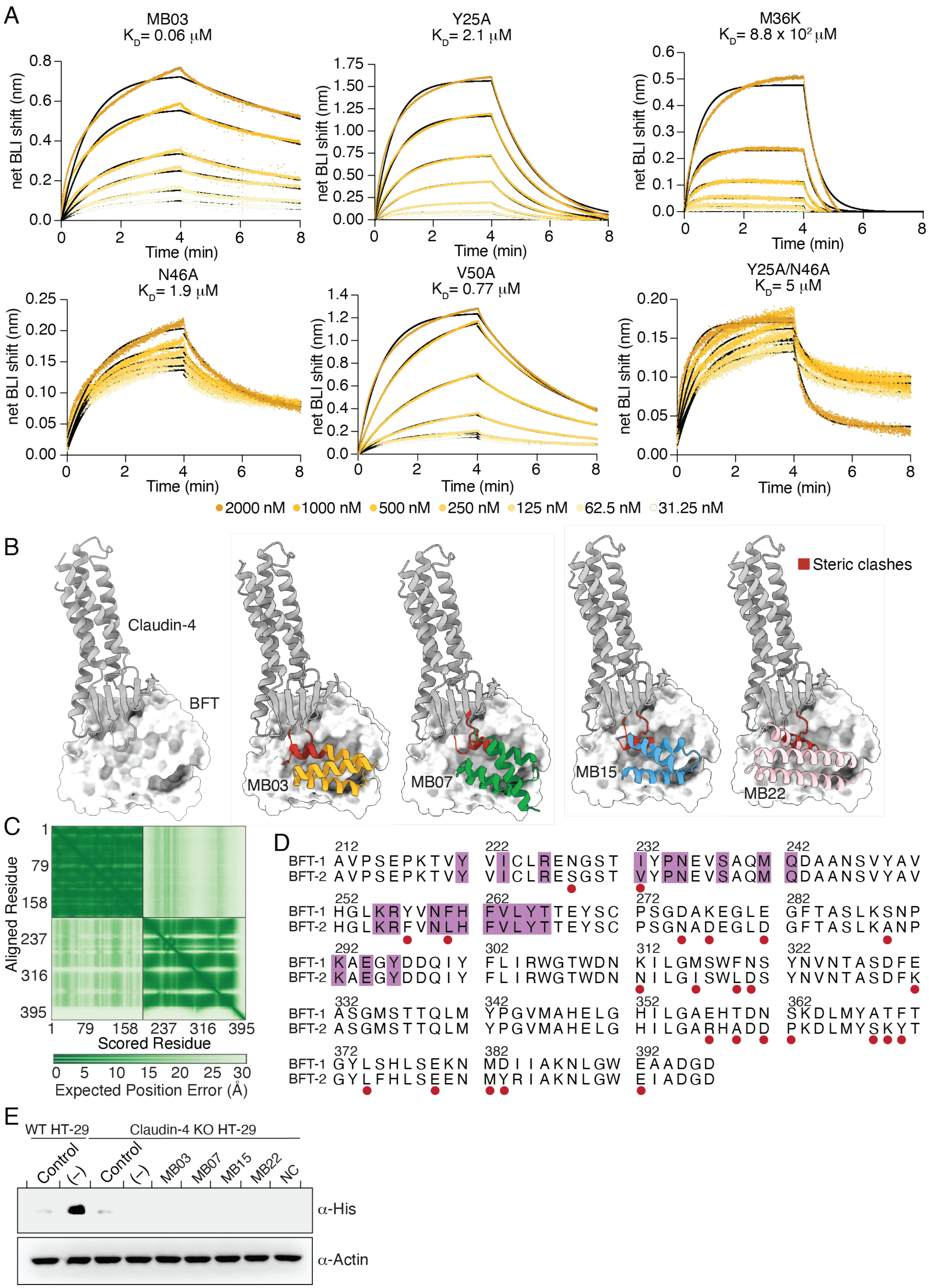

bioRxiv preprint doi: https://doi.org/10.64898/2026.06.22.733822; this version posted June 23, 2026. The copyright holder for this preprint (which was not certified by peer review) is the author/funder, who has granted bioRxiv a license to display the preprint in perpetuity. It is made available under a CC-BY 4.0 International license.

Supplemental Figure 9

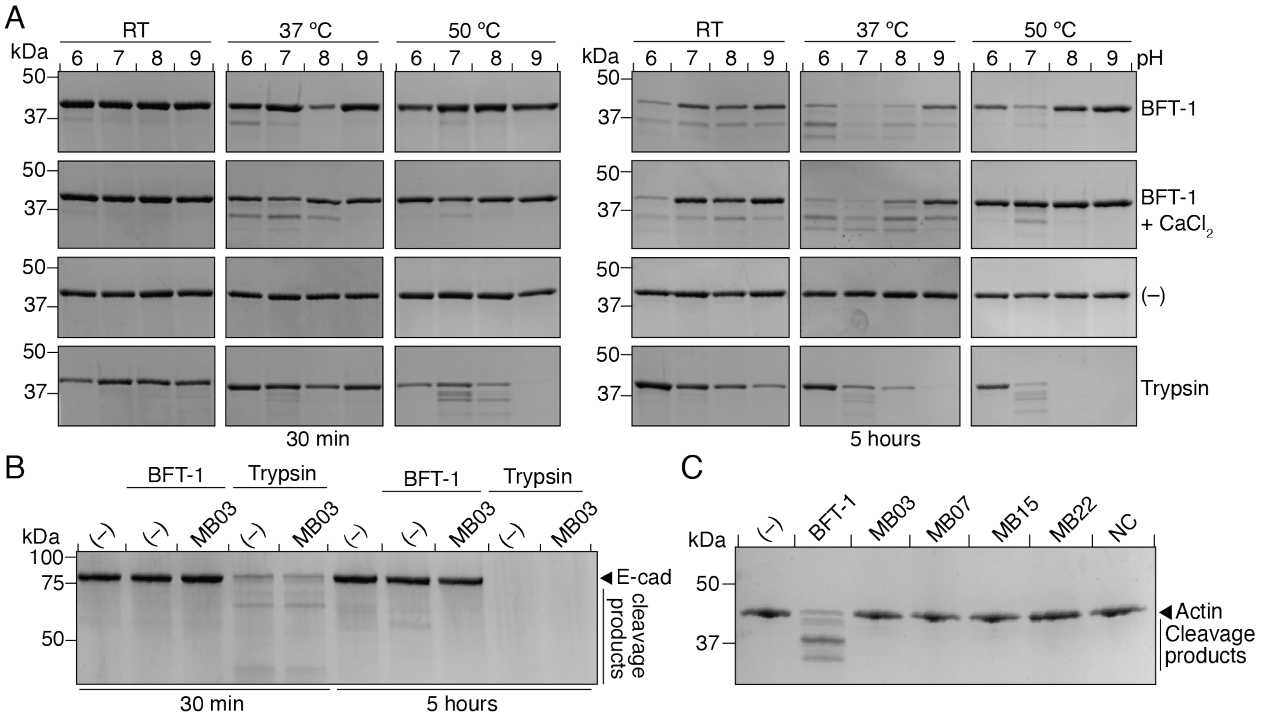

bioRxiv preprint doi: https://doi.org/10.64898/2026.06.22.733822; this version posted June 23, 2026. The copyright holder for this preprint (which was not certified by peer review) is the author/funder, who has granted bioRxiv a license to display the preprint in perpetuity. It is made available under a CC-BY 4.0 International license.

Supplemental Figure 10

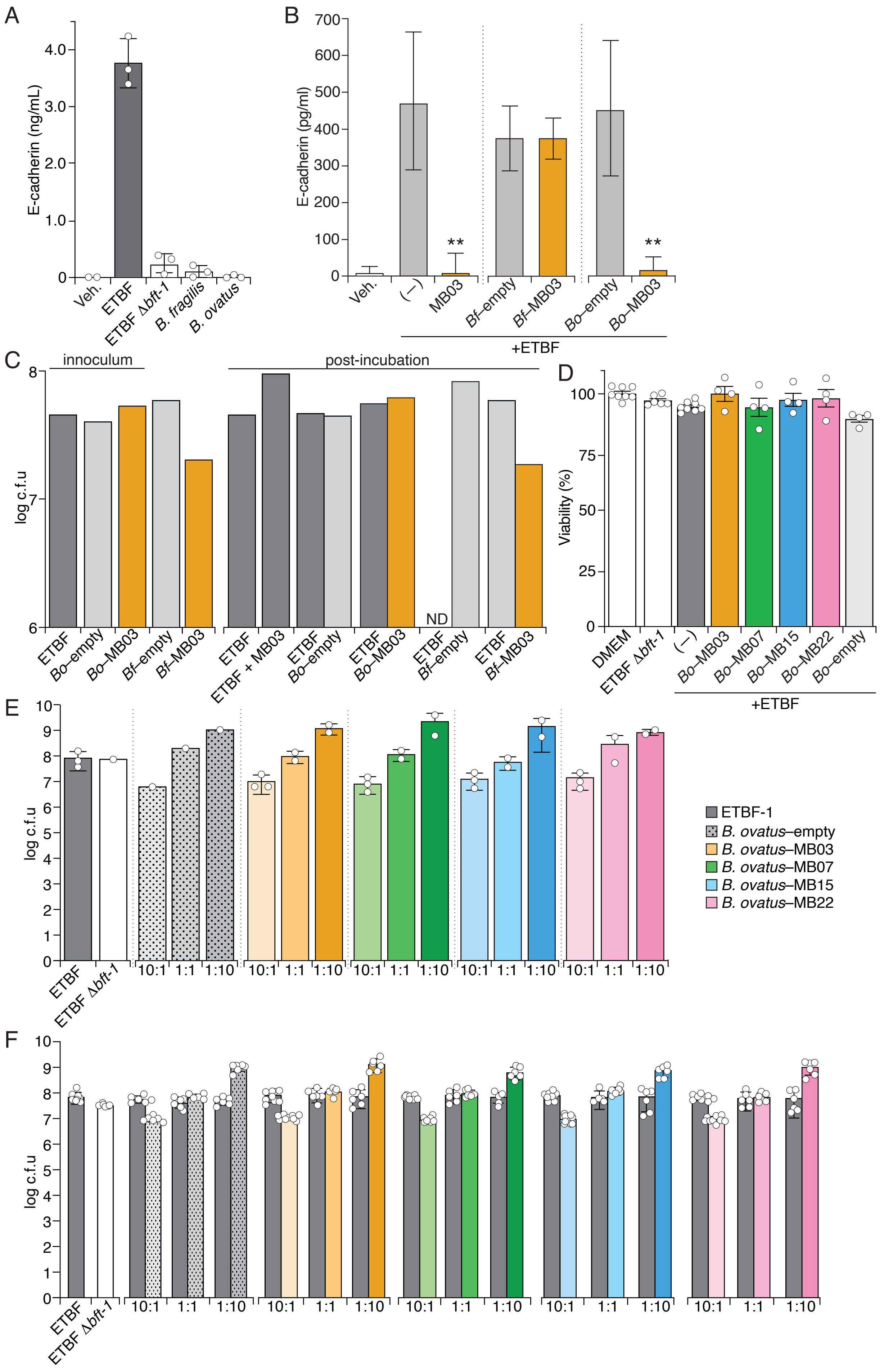

bioRxiv preprint doi: https://doi.org/10.64898/2026.06.22.733822; this version posted June 23, 2026. The copyright holder for this preprint (which was not certified by peer review) is the author/funder, who has granted bioRxiv a license to display the preprint in perpetuity. It is made available under a CC-BY 4.0 International license.

Supplemental Figure 11

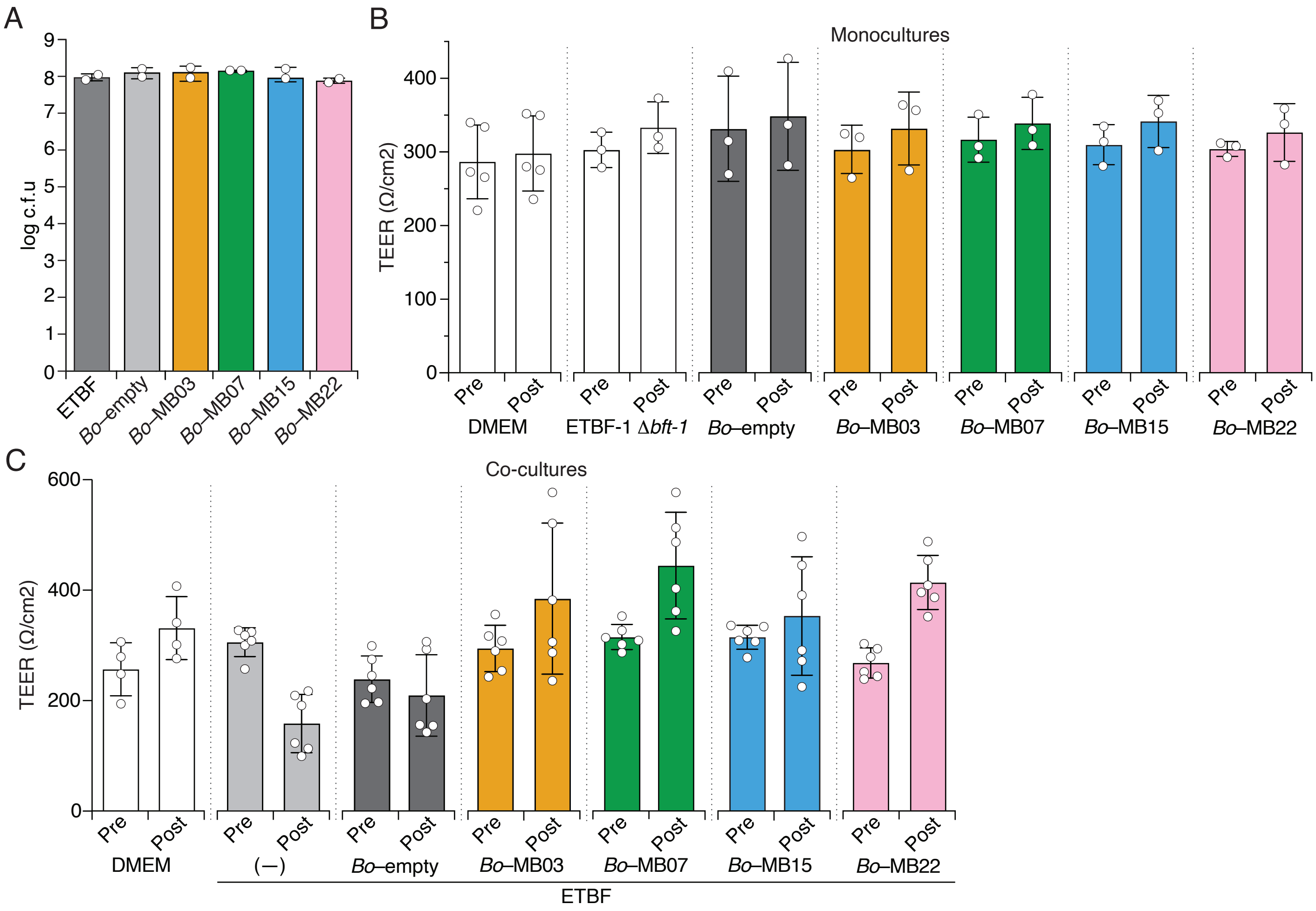

bioRxiv preprint doi: https://doi.org/10.64898/2026.06.22.733822; this version posted June 23, 2026. The copyright holder for this preprint (which was not certified by peer review) is the author/funder, who has granted bioRxiv a license to display the preprint in perpetuity. It is made available under a CC-BY 4.0 International license.

Supplemental Figure 12

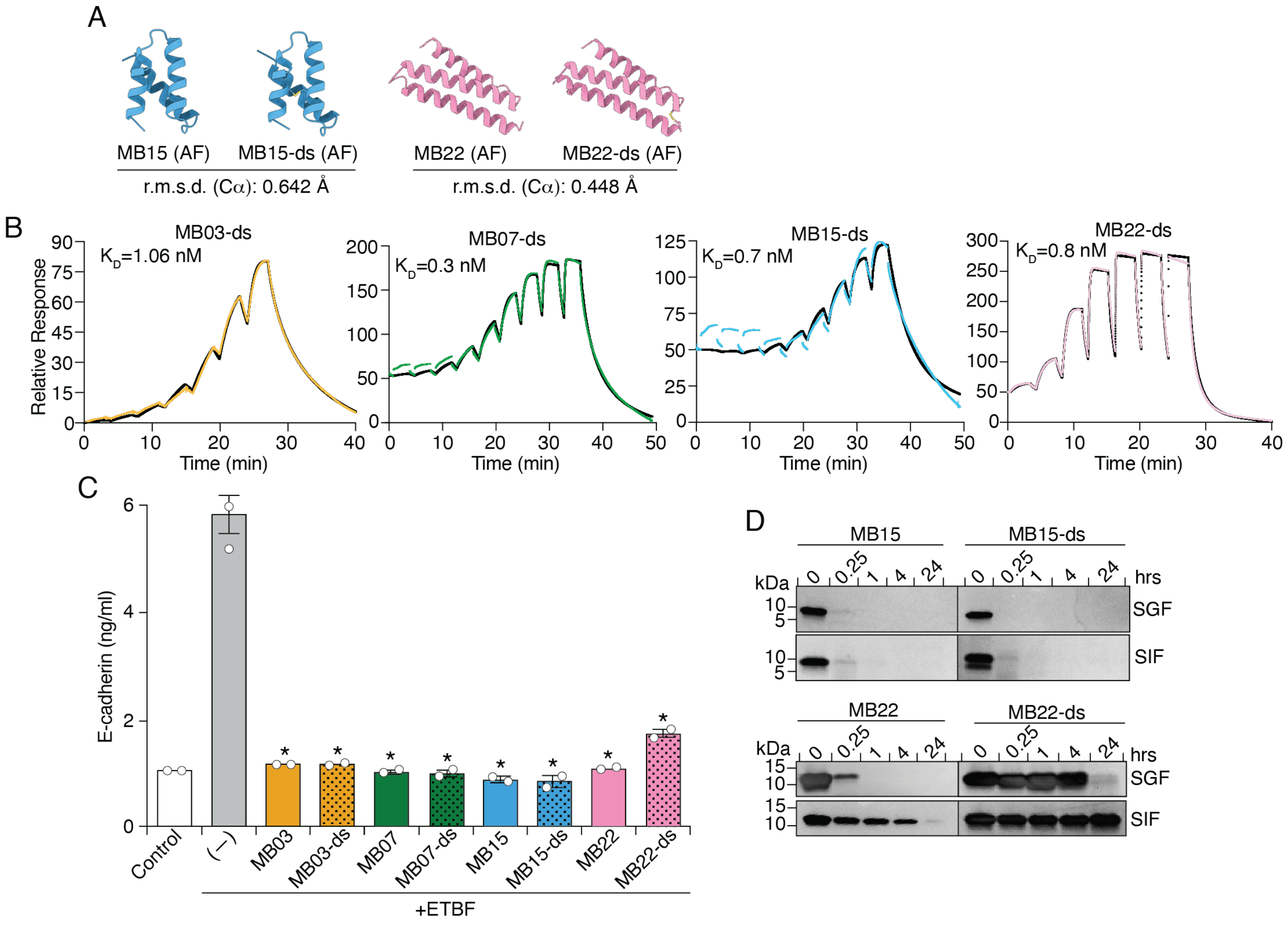

bioRxiv preprint doi: https://doi.org/10.64898/2026.06.22.733822; this version posted June 23, 2026. The copyright holder for this preprint (which was not certified by peer review) is the author/funder, who has granted bioRxiv a license to display the preprint in perpetuity. It is made available under a CC-BY 4.0 International license.

Supplemental Figure 13

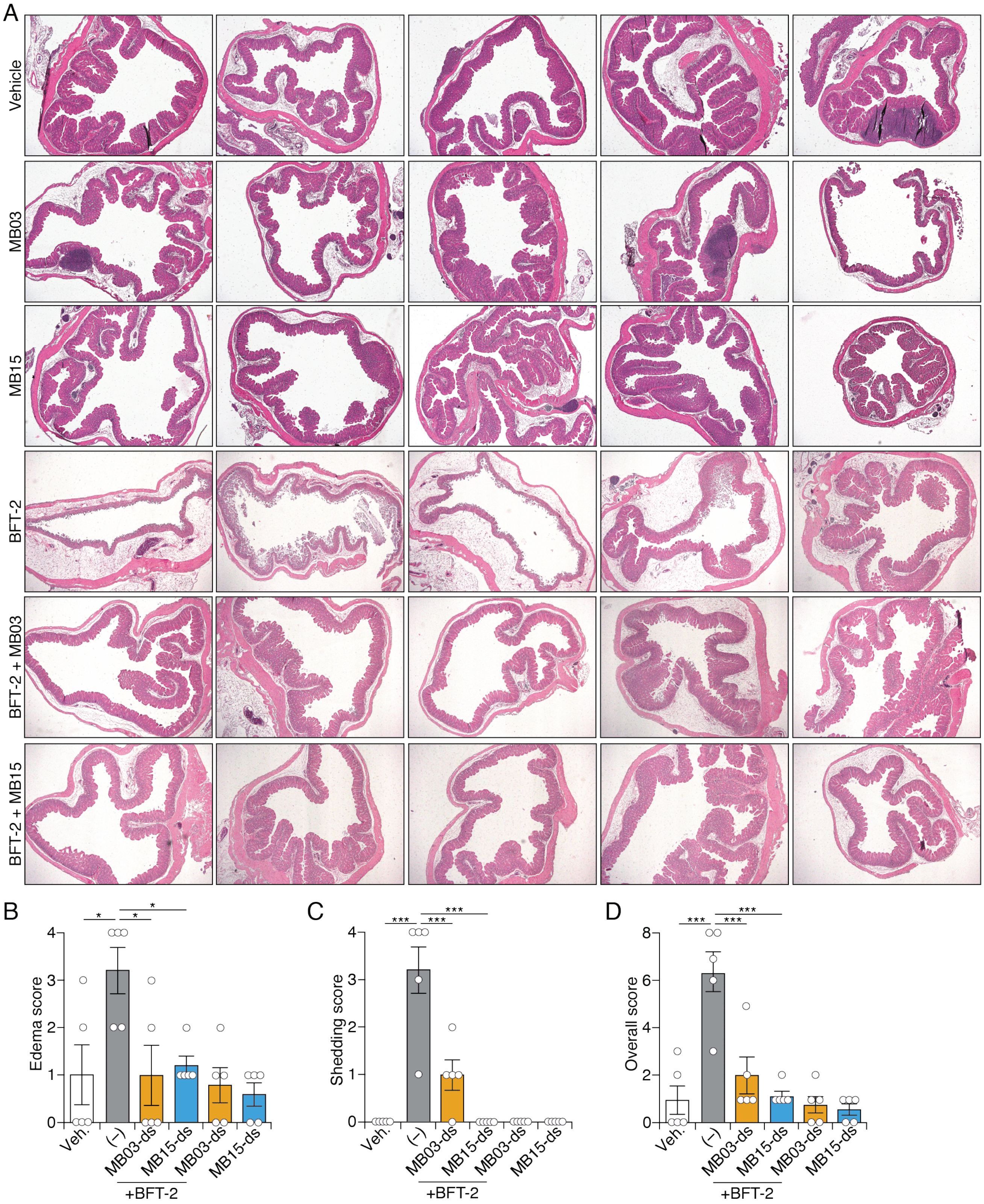

bioRxiv preprint doi: https://doi.org/10.64898/2026.06.22.733822; this version posted June 23, 2026. The copyright holder for this preprint (which was not certified by peer review) is the author/funder, who has granted bioRxiv a license to display the preprint in perpetuity. It is made available under a CC-BY 4.0 International license.

Supplemental Figure 14

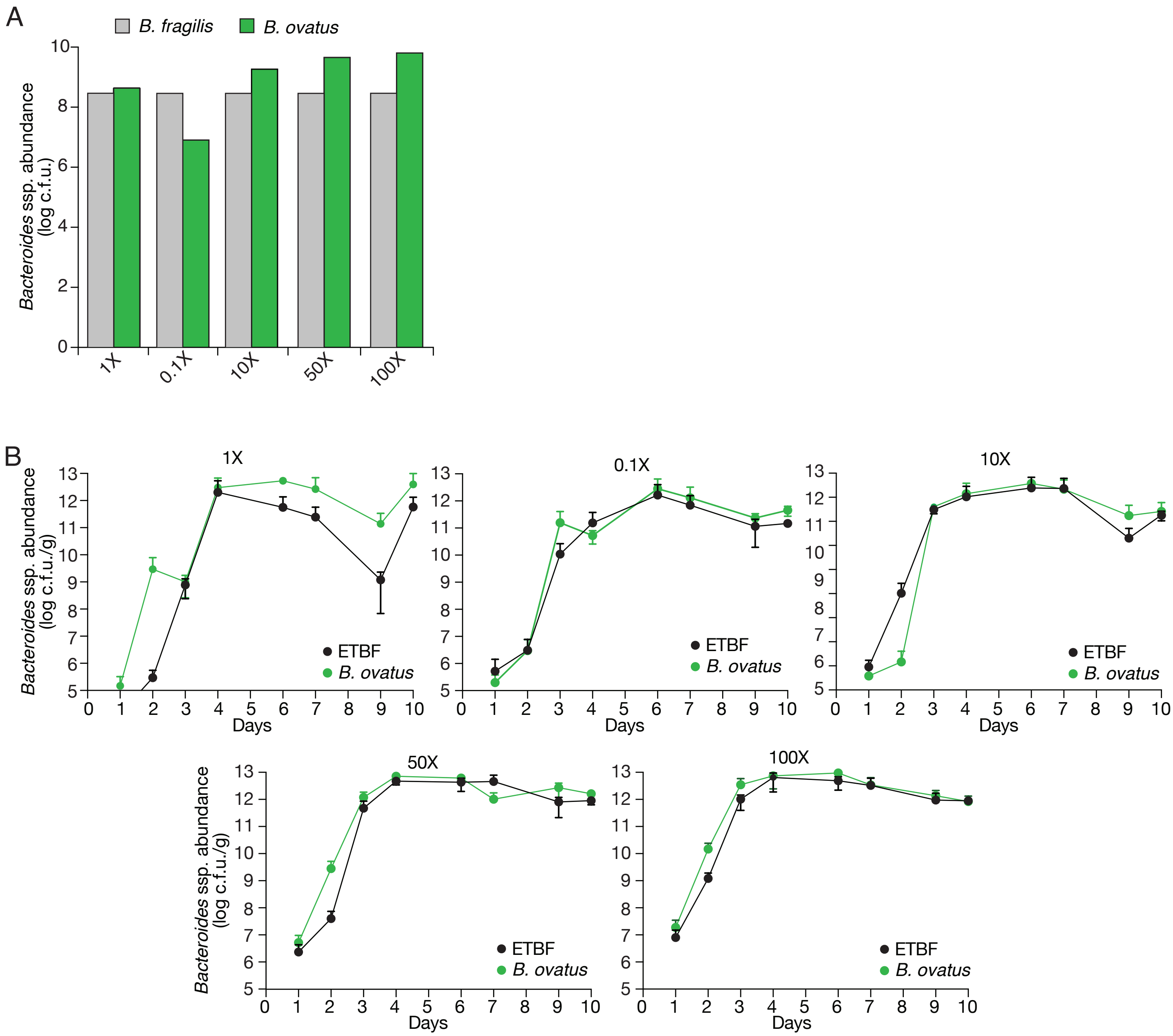

bioRxiv preprint doi: https://doi.org/10.64898/2026.06.22.733822; this version posted June 23, 2026. The copyright holder for this preprint (which was not certified by peer review) is the author/funder, who has granted bioRxiv a license to display the preprint in perpetuity. It is made available under a CC-BY 4.0 International license.

Supplemental Figure 15

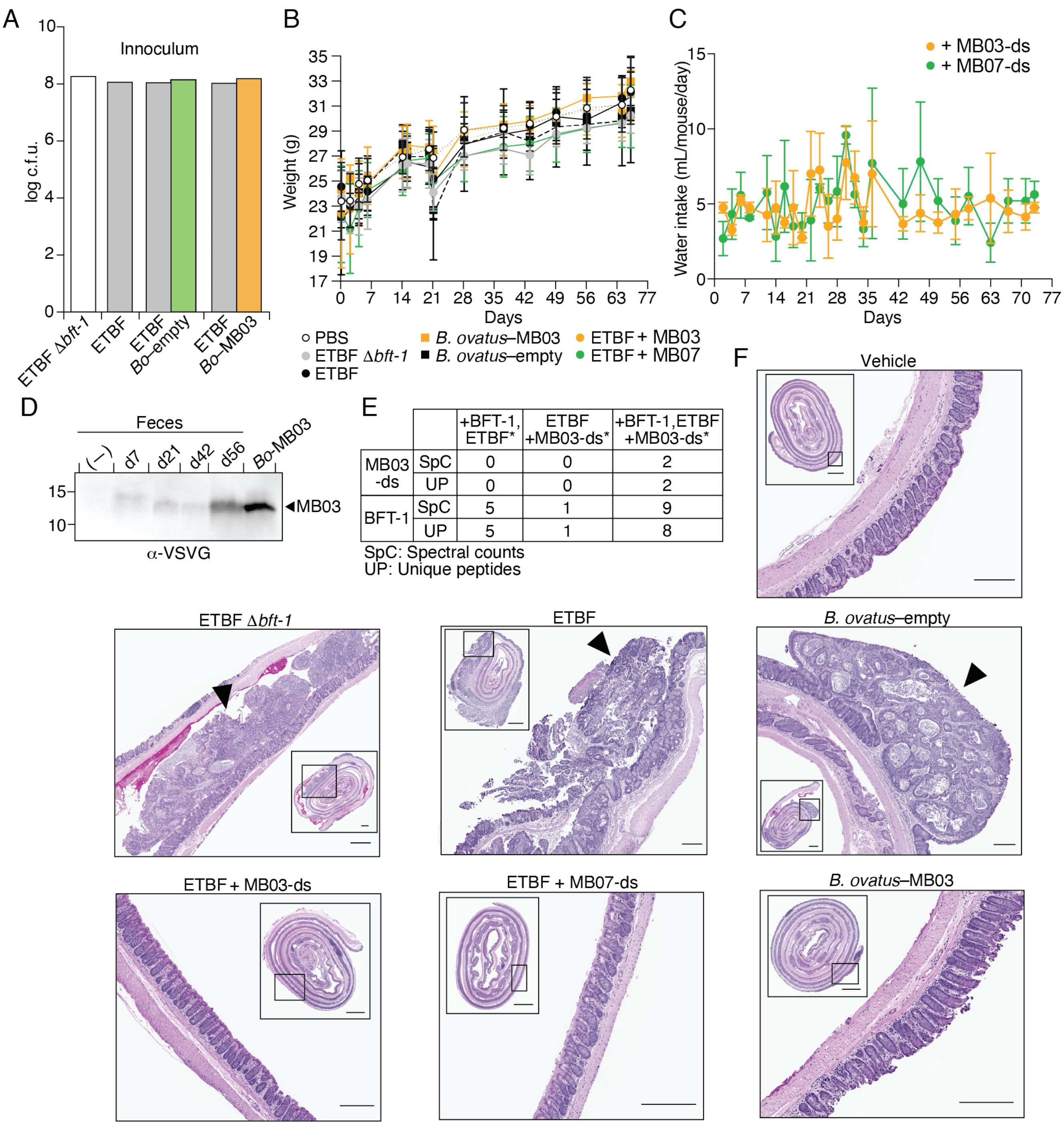

		+BFT-1, ETBF*	ETBF +MB03-ds*	+BFT-1,ETBF +MB03-ds*
MB03 -ds	SpC	0	0	2
	UP	0	0	2
BFT-1	SpC	5	1	9
	UP	5	1	8
